# Functional Limitations and Psychological Well-Being Among Older Adults in China: The Critical Role of *Hukou* as a Stratifying Factor

**DOI:** 10.1177/00914150221143997

**Published:** 2022-12-18

**Authors:** Jie Miao, Alex Bierman

**Affiliations:** Department of Sociology, 2129 University of Calgary, Calgary, Alberta, Canada

**Keywords:** stress process perspective, functional limitations, psychological well-being, *hukou*, China

## Abstract

Based on a stress process perspective, this study examines how the *hukou* system and gender intersect to shape the relationship between functional limitations and psychological well-being in older adults. Using the China Health and Retirement Longitudinal Study (*N* = 17,708 at baseline), analyses are carried out with random- and fixed-effects models. Analyses show that an urban *hukou* benefits the psychological well-being of Chinese older adults by weakening the relationship between functional limitations and depression, and these differences do not vary significantly between men and women. The relationship between functional limitations and life satisfaction does not differ by *hukou* region. This study shows that China's household registration system provides an important context for the associations between functional limitations and psychological well-being in later life. The *hukou* system is an important stratifying agent and should be taken into account in the study of stress and aging in a Chinese context.

Functional limitations are “restrictions in basic physical and mental actions” (Verbrugge & Jette, 1994, p. 4). Research suggests that functional limitations are substantial stressors among older adults, as functional limitations are significantly associated with a host of psychological outcomes in later life (Yang, 2006). In addition, longitudinal research suggests that prior levels of functional limitations predict changes in depressive symptoms over time (Gayman et al., 2013), demonstrating that functional limitations are a precipitating factor in decrements in psychological well-being (Pagulayan et al., 2008; Yang & George, 2005).

The association between functional limitations and the psychological well-being of older adults is especially important to examine in a Chinese context. China is experiencing unprecedented population aging (Chen & Liu, 2009; Yang & Du, 2021), which will lead more people in the nation than ever before to experience functional limitations. The burgeoning population of older adults with functional limitations will present tremendous demands on medical services and public support (Biritwum et al., 2016; Kämpfen et al., 2018). In addition, the psychological well-being of older adults has become an increasingly significant health issue in China in the past decades (Wang & Chen, 2014; Xu et al., 2018). However, due to historical and cultural factors, mental health services and care provision for older people are scarce, especially in rural areas (Kumar & Williams, 2021; Xiang et al., 2012; Yang & Tan, 2021). Greater attention is, therefore, needed to understand whether the increasing population preponderance of functional limitations is likely to exacerbate threats to the psychological well-being of older adults in China.

Sociological theory on psychological well-being underscores, however, that the psychological consequences of stress are not unitary. Stress process theory provides a paradigmatic sociological perspective on inequalities in psychological outcomes and particularly emphasizes that multiple social statuses often intersect to shape the effects of functional limitations (Pearlin & Bierman, 2013). An emphasis on the status-conditioned consequences of functional limitations is especially critical when studying older adults from China because of China's unique stratification system known as the *hukou* system. The *hukou* system is manifested in household registration barriers and different resource allocations between urban and rural areas (Chan & Buckingham, 2008; Chan & Wei, 2019). In this case, greater resources are provided to people born in an urban *hukou* (Brugiavini et al., 2018), suggesting that the *hukou* system may play a critical role in differentiating the consequences of functional limitations for psychological well-being among Chinese older adults. Moreover, research on functional limitations and psychological well-being underscores that multiple social statuses often intersect to shape the effects of functional limitations, and one of the most fundamental of these is gender (Bierman, 2012; Noh et al., 2016). Consequently, stress process theory leads to the question of how *hukou* status and gender may intersect to shape the ramifications of functional limitations for psychological well-being in Chinese older adults.

This research, therefore, has three main aims. The first is to replicate findings demonstrating deleterious consequences of functional limitations for psychological well-being in Western older adults in a national longitudinal study of Chinese older adults. Notably, the longitudinal nature of these data permits the application of fixed-effects models that holistically control for all time-stable sources of spuriousness (Allison, 2009), thereby providing an unusually robust test of this association within a Chinese context. The second is to show that China's unique *hukou* system plays a critical role in stratifying the psychological consequences of functional limitations. Third, we examine whether the moderating effects of the *rural–urban dual* system differ between men and women. This research, therefore, contributes to knowledge in the study of aging and psychological well-being by demonstrating the extent to which China's unique social context shapes the manifestation of the stress process and thereby differentiates the adverse psychological well-being effects of functional limitations late in life.

## Background

Functional limitations present as chronic stressors that exert both direct and indirect effects on psychological well-being (Brown, 2017; Yang, 2006). Functional limitations can challenge people's adaptive capacity and directly lead to the internal dysfunctions of an individual (Wheaton et al., 2013). Functional limitations may also influence psychological well-being by creating additional stressors in the process of “stress proliferation” (Pearlin, 1999), such as lost income and food insecurity (Heflin et al., 2019). The coexistence of functional limitations and additional stressors can create a compound burden on the psychological well-being of an individual (Yang, 2006; Young & Schieman, 2012). In addition, functional limitations may also reduce individuals’ sense of self and social support network, further leading to a detrimental impact on psychological well-being (Bierman, 2014; Yang, 2006). A substantial amount of research supports these arguments, showing a robust negative association between functional limitations and psychological well-being (Gayman et al., 2008). Research, however, has not commonly focused on examining this association in Chinese older adults. Moreover, while a few studies have found a positive association between dimensions of functional limitations and depression, this research has been cross-sectional in design (He et al., 2019; Li et al., 2019).

The reliance on cross-sectional analyses of the association between functional limitations and psychological well-being in Chinese older adults raises an added complication due to the degree to which this association may be overestimated due to time-stable sources of spuriousness. A spurious influence is a factor that affects both exposures to functional limitations and psychological well-being outcomes, thereby creating an appearance of an association between the two if this factor is not taken into account (Aneshensel, 2013). Relatively stable factors, such as personality traits and genetic predispositions, can influence exposure to both functional limitations and psychological well-being outcomes (Baek et al., 2016; Lamers et al., 2012; Leinonen et al., 2005), but it is rare to directly take these factors into account when examining the association between functional limitations and psychological well-being. In addition, research in a Western context underscores that a number of factors preceding later life can result in deleterious effects on functional status and mental well-being among older adults (Ferraro et al., 2016). China presents an especially critical context for lifetime adversities because Chinese older adults may have experienced a number of radical inequalities and social-economic transformations (Walder, 1989), and these factors could inflate the association between functional limitations and psychological well-being in later life. For example, experiencing the Great Famine in China has exhibited long-term influences on both functional limitations (Tao et al., 2019) and psychological well-being in later life (Li et al., 2018). Coming of age during the Cultural Revolution has also been linked to impaired physical and cognitive functioning in later life (Islam et al., 2017).

The totality of these personal, cultural, and structural challenges may critically spuriously inflate the association between functional limitations and psychological well-being, but their breadth also presents challenges for direct measurement as a means of statistical control. One alternative to attempts to measure and control for spurious influences is to use “fixed-effects” models, which hold constant time-stable sources of spuriousness, even when these factors are not directly observed (Allison, 2009). Analyses of Western samples, in fact, show that the association between functional limitations and psychological well-being is substantially reduced when these analyses are employed in the fixed-effects models (Bierman, 2012), but to our knowledge, this approach has not been employed in the study of Chinese older adults. In the current study, we, therefore, expect a deleterious association between functional limitations and measures of psychological well-being in Chinese older adults, but we also expect this association to weaken when time-stable sources of spuriousness are holistically controlled.

## Differentiation by *Hukou*

As the central component of China's rural–urban dual structure, the household registration system (also known as the *hukou* system) contributes to substantial socioeconomic differentiation between urban and rural residents (Chan & Wei, 2019). Since 1958, China has used the *hukou* system to assign each citizen a household registration identity based on the birthplace of his or her parents (Wang, 2010). People are issued with agricultural or non-agricultural *hukou* by the local government (Chan & Zhang, 1999; Mallee, 2003). An individual's *hukou* is not only related to familial area type but also, more importantly, to one's socioeconomic eligibility (Chan, 2009). Individuals who hold an agricultural/rural *hukou* survive on their own farming and harvesting, while individuals who hold a non-agricultural/urban *hukou* enjoy the social welfare provided by the government from birth (Liu, 2005). The *hukou* system not only determines the allocation of social benefits but also determines the distribution of other important resources such as education, housing, medical care, and employment (Kuang & Liu, 2012). One's *hukou* status follows the individual across the life course, with the result that most of the rural *hukou* holders cannot change their *hukou* to an urban one even if they migrate to urban areas (Chan, 2012). Although this system has evolved over time, the essential features of the *hukou* system remain unchanged and are still in effect today (Chan & Buckingham, 2008). Thus, the *hukou* system creates a social hierarchy that is in favor of individuals with an urban *hukou* by disproportionately allocating wealth, prestige, and power to urban *hukou* holders (Lui, 2016).

The stratification system that is constituted by the *hukou* system is relevant to the study of functional limitations and psychological well-being. The paradigmatic sociological perspective on stress and psychological well-being encapsulated in a “stress process perspective” suggests that inequalities in psychological well-being can be explained by the process of social stratification, through which power, prestige, and resources are unequally distributed to individuals in different social strata over time (Pearlin, 1999). Individuals occupying less advantaged placements in structures of social stratification are more vulnerable to the consequences of stressors due to restricted access to coping resources (Pearlin & Bierman, 2013). This perspective directs attention to the way that rural *hukou* holders are lacking in social and economic statuses (Kuang & Liu, 2012), and therefore, are more likely to be vulnerable to the deleterious effects of functional limitations on psychological well-being. The *hukou* system has played a key role in directing and allocating resources, thus creating a substantial gap in living conditions between urban and rural residents (Cheng & Selden, 1994), which will, in turn, amplify the effects of functional limitations in rural areas. *Hukou* registration has disproportionally allocated resources such as medical care, housing, and public services to urban *hukou* holders (Chan, 2019), and therefore, helped urban *hukou* alleviate the deleterious effects of the stressor. Having physical limitations may be more likely to cause secondary stressors for rural residents, such as loss of livelihood, as rural residents depend mainly on their own labor and the fluctuating harvests to survive (Liu, 2005). Rural residents may also have less access to health care and social services compared to their urban counterparts (Chan, 2009) and, therefore, may have inadequate services to aid in coping with the obstacles of daily living created by functional limitations. Thus, in the current study, we expect that *hukou* status shapes the psychological effects of functional limitations and rural *hukou* holders will experience more deleterious effects of functional limitations on psychological well-being than urban *hukou* holders.

## Gender Differences in the Moderating Effects of *Hukou*

A stress process perspective also calls attention to the ways that different social statuses intersect to shape the effects of stressors (Pearlin & Bierman, 2013). However, the intersectionality of different social statuses is often neglected in empirical research on functional limitations and psychological well-being. Moreover, gender has repeatedly been shown to be critical in differentiating the psychological consequences of functional limitations in previous research (Bierman, 2012; Noh et al., 2016). Building from this line of research, we suggest that the degree to which one's *hukou* status shapes the psychological consequences of functional limitations may differ between men and women.

Attention to the gendered context of *hukou* status is especially critical because there is a long history of gender inequality in China. Women are disadvantaged in China, as they are expected to work primarily from home, and therefore, do not have access to labor market resources (Hershatter, 2007; Ji et al., 2017), and are also denied community resources such as land possession, household ownership, and transportation (Li et al., 2008). This pattern of gender stratification also extends to the *hukou* system, in which women are less likely to benefit from the resources provided to urban *hukou* holders. In a rural *hukou*, women tend to have less favorable access to resources such as health care, *food*, and land compared to men (Murphy et al., 2011; Tian et al., 2018). Similarly, an urban *hukou* benefits men more than women, as women are underrepresented in senior positions in the labor market (Tatli et al., 2017), receive lower occupational pensions (Zhao & Zhao, 2018), and have smaller and less diverse social networks (Lin, 2000). This suggests that women in either an urban or rural *hukou* may experience disadvantages—in turn hindering the advantage of an urban *hukou* for women—with the result that the degree to which *hukou* status differentiates the psychological well-being effects of functional limitations is greater for men than women. In the current study, we, therefore, expect the degree to which *hukou* status shapes the association between functional limitations and psychological well-being to be stronger for men than for women.

## Methods

### Data

This study uses data from the China Health and Retirement Longitudinal Study (CHARLS), a nationally representative longitudinal survey of adults aged 45 and over in China. The sampling strategy is described in Zhao et al. (2014), from which this description is adapted. The baseline wave of the CHARLS was conducted between June 2011 and March 2012, surveying 17,708 respondents from 10,257 households (with a response rate of 80.5%), which included an assessment of the social, economic, and health circumstances of community residents. Participants were randomly sampled using a “multi-stage probability-proportional-to-size technique (PPS)” (Zhao et al., 2014, p. 62). In the first stage, 150 county-level units were randomly chosen from a sampling frame that contains all county-level units in China. After that, three primary sampling units (PSUs) were selected within each county-level unit. The CHARLS used the lowest level of the government organization (i.e., villages in rural areas and neighborhoods in urban areas) as PSUs. The final sample contains 450 PSUs which fell within 28 provinces, with the exception of Tibet. We utilized two additional follow-up surveys spaced two years apart. The respondent attrition rate between wave 1 and wave 2 is 14.24%, and the respondent attrition rate between wave 1 and our third observation point is 17.69%. Techniques used to address survey attrition and the clustered nature of the sample will be addressed below.

### Focal Measures

*Psychological Well-being*. Research in the sociology of psychological well-being emphasizes the importance of studying a diverse set of outcomes to fully appreciate the contours of the consequences of stress for psychological well-being (Aneshensel, 2005). In particular, research on the consequences of negative events suggests that positive and negative psychological outcomes may not exhibit coordinated changes (Diener et al., 2009). As emphasized by previous aging research, examining both life satisfaction and symptoms of depression can “more fully represent underlying levels of psychological well-being than would be demonstrated through only one indicator” (Lee et al., 2020, p. 2210). Therefore, within this research, we follow the approach of examining symptoms of depression as a negative psychological outcome and life satisfaction as a positive psychological outcome.

Symptoms of depression at each wave were measured using a 10-item Center for Epidemiologic Studies Depression Scale (CESD-10) that has been validated for use in Chinese older adults (Chen & Mui, 2014). Respondents were asked to rate depressive symptoms in the past week on a four-point scale from occurring rarely or none of the time to most or all of the time. Each of the symptoms is listed in Table 1. With two items reverse-coded, the sum of the CESD-10 scores ranged from 0 to 30, with higher scores indicating greater depressive symptoms.

**Table 1.** Symptoms of Depression.

Symptoms of depression
1. I was bothered by things that do not usually bother me.
2. I had trouble keeping my mind on what I was doing.
3. I felt depressed.
4. I felt that everything I did was an effort.
5. I felt hopeful about the future.
6. I felt fearful.
7. My sleep was restless.
8. I was happy.
9. I felt lonely.
10. I felt I could not “get going”.

Life satisfaction was measured by a single item—“how satisfied are you with your life-as-a-whole?”—rated on a five-point scale, ranging from 1 (completely satisfied) to 5 (not at all satisfied). The item was recoded so that higher scores correspond to higher life satisfaction. The validity of a single-item measure of life satisfaction has been supported through a comparison to a multiple-item measurement of life satisfaction (Cheung & Lucas, 2014).

*Functional limitations*. Functional limitations at each wave were measured by difficulty in regularly performing seven general physical activities: walking 100 m, climbing several flights of stairs, getting up from a chair, stooping, or kneeling, or crouching, extending arms up, picking up a small coin, and lifting ten jin (equivalent to 5 kg). The functional limitation scale is constructed by summing the values of the seven items on which difficulty was reported. Higher scores reflect more difficulties in performing mobility activities.

*Gender.* Gender is coded as a dichotomous variable, so that 1 = women and 0 = men.

*Hukou*. We used respondents’ reports of current *hukou* status in wave 1 to classify them as rural (agricultural) or urban (non-agricultural) residents. *Hukou* is coded as a dichotomous variable so that 1 = urban (non-agricultural) residents and 0 = rural (agricultural) residents.

### Control Measures

We include a common set of demographic controls that have been shown to be associated with psychological well-being in Chinese older adults (Li et al., 2019). Age is coded in years. Familial social support resources were taken into account, with controls for the number of people in the household and marital status at each wave. Marital status at each wave is coded as a dichotomous variable, in which 0 = widowed, divorced, separated, or never married and 1 = married or partnered. *Socioeconomic status (SES)* was controlled using education at baseline, work status, and income at each wave. Education was measured by the highest level of school completed, and the 12 responses were grouped into five categories—illiterate, elementary school, junior high school, senior high school, and college or more, using illiterate as a reference. Work status was measured based on a set of dichotomous variables at each wave—agricultural work, non-agricultural work, and do not work—with agricultural work as a reference. Income was measured as a dichotomous variable, in which 0 = total annual income above the poverty line for the year and 1 = total annual income below the poverty line for the year.^1^ To take into account other health conditions that may influence psychological well-being, we controlled for self-rated health at each wave using a set of dichotomous indicators—good/very good, fair, and poor/very poor for three waves—using good and very good as the reference. Table 2 presents sample characteristics for the focal and control measures.

**Table 2.** Descriptive Statistics.

	Time 1		Time 2		Time 3	
	x̄	S	x̄	S	x̄	S
Time-varying measures						
Functional limitations	1.190	1.560	1.408	1.688	1.501	1.743
Depression	8.033	6.168	7.656	5.676	7.929	6.322
Life satisfaction	3.054	0.709	3.109	0.739	3.374	0.773
Work status						
Non-agricultural work	0.285	0.451	0.260	0.438	0.263	0.440
Not work	0.407	0.491	0.392	0.488	0.414	0.493
Partnered	0.856	0.351	0.848	0.359	0.838	0.368
Number of people in the household	3.593	1.656	3.625	1.658	3.036	1.305
Self-rated health						
Good self-rated health	0.256	0.436	0.229	0.420	0.229	0.420
Poor self-rated health	0.264	0.441	0.245	0.430	0.231	0.421
Low income	0.209	0.406	0.181	0.385	0.166	0.372
Time-stable measures						
Urban *hukou*	0.290	0.454				
Women	0.524	0.499				
Education						
Elementary school	0.213	0.410				
Junior high school	0.212	0.409				
Senior high school	0.089	0.284				
Post-secondary school	0.064	0.244				
Baseline age	58.776	10.730				

*Note. N* = 17,708. Descriptives are weighted and calculated through full information maximum likelihood estimation.

### Plan of Analysis

Analyses are carried out using a combination of random- and fixed-effects models, estimated through the structural equation model (SEM). A random-effects model is useful in longitudinal analyses by adjusting standard errors for repeated observations but can only rule out spuriousness by controlling the observed covariates (Wooldridge, 2015). Conversely, a fixed-effects model controls for all time-stable sources of confounding, even if these confounders are not directly observed (Allison, 2009). One drawback to conventional fixed-effects models is that time-stable predictors cannot be included in the model due to being colinear with the control for all time-stable influences, but this weakness can be addressed through a hybrid model estimated through SEM (Bollen & Brand, 2010). In the SEM approach, a set of outcome measures at each wave are used as indicators of a latent variable that represents all time-stable influences on the outcome (Allison, 2009). In a random-effects model, this latent variable is not allowed to correlate with the predictors, while this latent variable is allowed to correlate with observed predictors in the fixed-effects estimates. The hybrid model, therefore, estimates random-effects model coefficients for time-stable predictors by disallowing the correlation between the latent variable and time-stable predictors and fixed-effects model coefficients for time-varying predictors by allowing the latent variable to correlate with these predictors.

An additional advantage of the use of SEM to estimate random and fixed-effects models is that SEM provides multiple model fit indices. Primary among these is the chi-square statistic, which provides an overall measure of model fit that can be used to compare nested models to assess improvement in model fit (Kline, 2005). The chi-square statistic can also be used to test the fit of a single model, with a non-significant chi-square being preferred, but due to the tendency for a significant chi-square in larger sample sizes, alternative measures of model fit are preferred when examining a single model's fit (Byrne, 2012). These include the comparative fit index (CFI) and the root mean square error of approximation (RMSEA), with values of 0.95 or greater preferred for the CFI and 0.06 or less for the RMSEA (Byrne, 2012).

All models are estimated using Stata 15.2. Models are estimated using a “full information” maximum likelihood (FIML) estimator. FIML addresses missing data by utilizing all information available from each case, thereby providing less biased analyses compared with more conventional missing data methods (e.g., listwise deletion and mean imputation) (Allison, 2003; Enders, 2010). All analyses use the Taylor series estimation of standard errors, which adjust standard errors for the multi-stage sampling of villages/neighborhoods and respondents (Heeringa et al., 2017). Sampling weights are also applied in the analyses, but the measures of model fit are based on unweighted analyses, due to Stata's application of pseudo-log likelihood when weights are utilized.

For each outcome, analyses are carried out in a series of steps. We first examine the association between functional limitations and psychological well-being in a random-effects model that includes all observed controls. A second model is a hybrid model, which estimates fixed-effects model coefficients for functional limitations. The second model will serve to demonstrate the extent to which unobserved time-stable factors bias the estimation of these relationships, even after commonly observed controls are included in the model. A third model then introduces the time-varying controls (while allowing the latent variable to correlate with these predictors as well) to demonstrate the extent to which the association between functional limitations and psychological well-being is further reduced due to time-varying sources of spuriousness. Following this, we test an interaction between *hukou* and functional limitations to examine whether *hukou* moderates the relationship between functional limitations and psychological well-being. We then test a three-term interaction between functional limitations, gender, and *hukou* to examine whether the moderating effect of *hukou* differs by gender.

## Results

### Functional Limitations and Depression

Table 3 presents the results of the models of symptoms of depression. Model 1 is a random-effects model that adjusts standard errors for repeated observations and includes time-stable controls. This model shows that there is a significant positive association between functional limitations and depression (*p* < 0.001), indicating that a greater number of functional limitations is associated with higher levels of depression. This relationship is also relatively substantial—a one-unit increase in functional limitations is related to a 1.25-unit increase in the level of depression; when standardized using baseline variances, this association was 0.32. However, this model does not control for unobserved time-stable confounds that may influence the relationship between functional limitations and depression. The CFI for this model also does not indicate adequate model fit, with a value below 0.95, although the fit indicated by the RMSEA is acceptable.

**Table 3.** Functional Limitations and Depression.

	Model 1			Model 2			Model 3			Model 4			Model 5		
	*b*	*SE*		*b*	*SE*		*b*	*SE*		*b*	*SE*		*b*	*SE*	
Functional limitations	1.246	0.030	***	0.793	0.036	***	0.703	0.035	***	0.750	0.037	***	0.786	0.054	***
Urban *hukou*	−1.161	0.179	***	−1.270	0.180	***	−1.190	0.178	***	−0.916	0.179	***	−0.684	0.183	***
Women	0.813	0.077	***	1.024	0.080	***	0.779	0.080	***	0.777	0.081	***	0.942	0.128	***
Elementary school^a^	−0.668	0.121	***	−0.785	0.128	***	−0.722	0.125	***	−0.698	0.126	***	−0.683	0.127	***
Junior high school^a^	−1.011	0.148	***	−1.118	0.155	***	−1.020	0.156	***	−1.001	0.160	***	−0.976	0.160	***
Senior high school^a^	−1.532	0.182	***	−1.704	0.185	***	−1.518	0.191	***	−1.520	0.191	***	−1.493	0.194	***
Post-secondary school^a^	−1.773	0.269	***	−1.993	0.265	***	−1.627	0.258	***	−1.656	0.256	***	−1.658	0.255	***
Age (years)	−0.019	0.006	**	0.009	0.007		−0.024	0.008	**	−0.023	0.008	**	−0.023	0.008	**
Partnered							−1.393	0.278	***	−1.386	0.276	***	−1.387	0.277	***
Non-agricultural work^b^							−0.227	0.113	*	−0.233	0.113	*	−0.236	0.113	*
Not work^b^							0.203	0.122		0.197	0.122		0.195	0.122	
Number of people in the household							−0.075	0.036	*	−0.075	0.036	*	−0.075	0.036	*
Good self-rated health^c^							−0.697	0.073	***	−0.700	0.073	***	−0.700	0.073	***
poor self-rated health^c^							1.381	0.102	***	1.378	0.103	***	1.376	0.103	***
Low income							0.164	0.092		0.162	0.092		0.161	0.092	
Functional limitations × Urban *hukou*										−0.232	0.092	*	−0.323	0.142	*
Functional limitations × Women													−0.056	0.069	
Urban *hukou* × Women													−0.464	0.282	
Functional limitations × Urban *hukou* × Women													0.153	0.187	
Intercept_T1_	8.142	0.425	***	7.071	0.465	***	10.350	0.632	***	10.189	0.654	***	10.085	0.652	***
Intercept_T2_	7.523	0.414	***	6.559	0.456	***	9.834	0.627	***	9.683	0.648	***	9.577	0.645	***
Intercept_T3_	7.662	0.411	***	6.755	0.453	***	9.981	0.622	***	9.824	0.645	***	9.719	0.642	***
R^2^_T1_	0.473			0.504			0.518			0.518			0.518		
R^2^_T2_	0.545			0.571			0.571			0.572			0.572		
R^2^_T3_	0.481			0.504			0.514			0.515			0.514		
χ^2^	1175.953		***	245.443		***	329.014		***	338.396		***	347.905		***
*df*	24.000			21.000			56.000			61.000			73.000		
Comparative fit index	0.938			0.988			0.988			0.988			0.988		
Root mean square error of approximation	0.043			0.02			0.014			0.013			0.012		

*Notes.* Metric coefficients are presented. *N* = 17,708. SE = standard error.

^a^ Illiterate is the reference group.

^b^ Agricultural work is the reference group.

^c^ Fair self-rated health is the reference group

*p < 0.05, **p < 0.01, ***p < 0.001 (two-tailed tests).

Model 2 creates a hybrid model by allowing the latent indicator of unobserved time-stable characteristics to covary with the time-stable predictors, thereby ruling out all sources of spuriousness due to time-stable confounds in the estimation of the association between depression and the time-stable predictors. The change in the chi-square statistics between Model 1 and Model 2 is significant (Δχ^2^ = 930.51, df = 3, *p* *< 0.001*), indicating that allowing these covariances significantly improves model fit. Moreover, both the CFI and RMSEA now indicate an acceptable model fit. A model that controls for all time-stable sources of spuriousness is therefore preferred over the random-effects model. When these additional time-stable influences are controlled, the coefficient for the relationship between functional limitations and depression decreases from Model 1 by 36% but still remains significant at *p < 0.001*. This reduction suggests that more than a third of the association between functional limitations and depression in Model 1 is spurious due to unobserved time-stable confounders. Supplemental analyses based on baseline variances showed that the standardized association between functional limitations and depression in Model 2 was reduced to 0.20, still indicating a substantial association once time-stable sources of confounding are holistically controlled.

Model 3 introduces a vector of time-varying controls. The latent measure of time-stable influences is allowed to correlate with these predictors as well, with the result that estimates of the associations between depression and all time-varying predictors are also absent of time-stable sources of spuriousness. Model 3 shows that the association between functional limitations and depression is reduced from Model 2 by 11% when the vector of observed time-varying controls is included in the model, and the association remains significant at *p < 0.001*. The relatively small reduction in Model 3 suggests that time-stable influences are the major source of spuriousness in the association between functional limitations and depressive symptomology.

Although the prior analyses examine the association between functional limitations and depression, they do not show whether this association differs by *hukou*. Model 4 includes an interaction between *hukou* status and functional limitations. This interaction is significant, indicating that the relationship between *functional limitations* and depression varies significantly between urban and rural *hukou* holders. Furthermore, the coefficient for this interaction is negative, indicating that the deleterious association between functional limitations and depression is weaker among older adults living in an urban *hukou* as compared to those living in a rural *hukou*.

**Figure 1.**
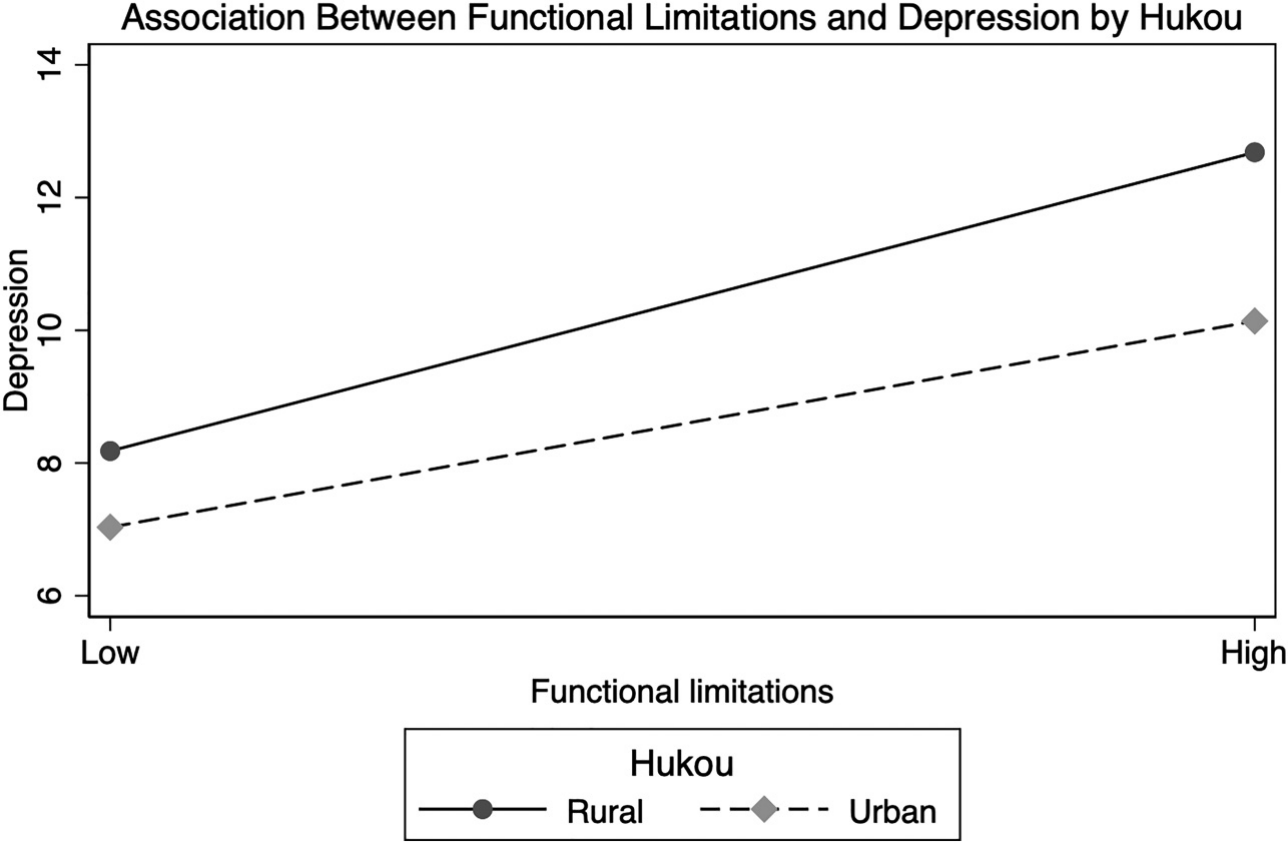
Association between functional limitations and depression by Hukou.

Figure 1 shows the predicted values for the association between functional limitations and depression separately by *hukou* status, with all controls held constant at their respective means. A steeper slope for rural *hukou* holders indicates that the association between functional limitations and depression is stronger among older adults with a rural *hukou* status. Ancillary analyses showed that the slope for functional limitations is significant for both urban and rural hukou status. This indicates that an urban *hukou* buffers the effects of functional limitations on depressive symptomology, but an urban *hukou* does not entirely eliminate the deleterious association between functional limitations and symptoms of depression.

However, these analyses do not indicate whether the buffering effects of *hukou* differ by gender. Model 5 tests a three-term interaction between *hukou* region, gender, and functional limitations. This interaction is not significant *(p = 0.415)*, indicating that there are no significant differences between men and women in the moderating effects of *hukou* status on the association between functional limitations and symptoms of depression.

### Functional Limitations and Life Satisfaction

Table 4 presents the results of the models of life satisfaction. Model 1 is a random-effects model of life satisfaction that includes the observed time-stable controls. This model shows a significant negative association between functional limitations and life satisfaction, indicating that greater functional limitations are associated with lower levels of life satisfaction. The coefficient for functional limitations and life satisfaction is -0.07 and is significant at *p < 0.001*. When standardized using baseline variances, this association is -0.15, indicating a weaker association than for depression.

**Table 4.** Functional Limitations and Life Satisfaction.

	Model 1			Model 2			Model 3			Model 4			Model 5		
	*b*	*SE*		*b*	*SE*		*b*	*SE*		*b*	*SE*		*b*	*SE*	
Functional limitations	−0.067	0.004	***	−0.041	0.004	***	−0.033	0.004	***	−0.034	0.005	***	−0.031	0.009	***
Urban *hukou*	0.005	0.022		0.012	0.021		0.005	0.023		−0.001	0.025		−0.027	0.031	
Women	0.015	0.015		0.003	0.015		0.018	0.014		0.018	0.014		0.010	0.021	
Elementary school^a^	−0.034	0.025		−0.027	0.025		−0.027	0.025		−0.027	0.025		−0.030	0.025	
Junior high school^a^	0.000	0.018		0.006	0.018		−0.003	0.018		−0.004	0.018		−0.007	0.018	
Senior high school^a^	−0.046	0.039		−0.037	0.040		−0.060	0.041		−0.060	0.041		−0.063	0.041	
Post-secondary school^a^	0.043	0.030		0.056	0.030		0.024	0.028		0.024	0.028		0.025	0.028	
Age (years)	0.009	0.001	***	0.007	0.001	***	0.009	0.001	***	0.009	0.001	***	0.009	0.001	***
Partnered							0.034	0.039		0.034	0.039		0.035	0.039	
Non-agricultural work^b^							0.042	0.017	*	0.042	0.017	*	0.042	0.017	*
Not work^b^							0.004	0.017		0.004	0.017		0.004	0.017	
Number of people in the household							0.012	0.005	*	0.012	0.005	*	0.012	0.005	*
Good self-rated health^c^							0.142	0.016	***	0.142	0.016	***	0.142	0.016	***
poor self-rated health^c^							−0.082	0.014	***	−0.082	0.014	***	−0.082	0.014	***
Low income							−0.028	0.013	*	−0.028	0.013	*	−0.028	0.013	*
Functional limitations × Urban *hukou*										0.005	0.010		0.005	0.017	
Functional limitations × Women													−0.005	0.011	
Urban *hukou* × Women													0.054	0.036	
Functional limitations × Urban *hukou* × Women													−0.002	0.024	
Intercept_T1_	2.608	0.056	***	2.668	0.059	***	2.466	0.089	***	2.468	0.089	***	2.474	0.089	***
Intercept_T2_	2.679	0.057	***	2.734	0.060	***	2.534	0.089	***	2.537	0.089	***	2.542	0.089	***
Intercept_T3_	2.952	0.055	***	3.003	0.057	***	2.808	0.085	***	2.810	0.086	***	2.816	0.085	***
R^2^_T1_	0.339			0.343			0.349			0.349			0.349		
R^2^_T2_	0.325			0.331			0.341			0.341			0.341		
R^2^_T3_	0.285			0.289			0.296			0.295			0.296		
χ^2^	231.199		***	85.070		***	165.417		***	169.737		***	185.764		***
*Df*	24.000			21.000			56.000			61.000			73.000		
Comparative fit index	0.955			0.986			0.983			0.984			0.983		
Root mean square error of approximation	0.018			0.011			0.009			0.008			0.008		

*Notes.* Metric coefficients are presented. *N* = 17,708. SE = standard error.

^a^ Illiterate is the reference group.

^b^ Agricultural work is the reference group.

^c^ Fair self-rated health is the reference group

*p < 0.05, **p < 0.01, ***p < 0.001 (two-tailed tests).

Although the CFI and RMSEA show acceptable model fit for Model 1, Model 2 shows improved model fit by allowing the latent measure of unobserved time-stable influences to covary with the time-varying predictors. Between Model 1 and Model 2, the CFI increases from 0.955 to 0.986, and the RMSEA drops from 0.018 to 0.011. More importantly, the change in the chi-square statistics between Model 1 and Model 2 is significant (Δχ^2^ = 146.13, df = 3, *p* *< 0.001*), indicating that model fit is significantly improved by controlling for unobserved time-stable influences, in turn demonstrating that a fixed-effects model is preferable to a random-effects model. In Model 2, the coefficient for the relationship between functional limitations and life satisfaction decreases by 39% from Model 1, indicating that a substantial portion of the association between functional limitations and life satisfaction is spurious due to unobserved time-stable confounders. Although the remaining association with functional limitation is significant at *p < 0.001*, supplementary analyses using baseline variances showed that the standardized coefficient for functional limitations and life satisfaction is approximately 0.09. With time-stable sources of confounding taken into account, then there is a relatively weak overall association between functional limitations and life satisfaction in Chinese older adults.

Model 3 includes a vector of time-varying controls. The latent measure of time-stable influences is allowed to correlate with these predictors, with the result that the estimates of the association between these predictors and life satisfaction control for all time-stable sources of confounding. The association between functional limitations and life satisfaction is reduced by 20% when the vector of observed time-varying controls is included in Model 3, but this relationship remains significant at *p < 0.001*. When standardized based on baseline variances, the association between functional limitations and life satisfaction is reduced to 0.07. There is, therefore, only a weak association between functional limitations and life satisfaction once time-stable and time-varying sources of spuriousness are taken into account.

Model 4 includes an interaction between *hukou* status and functional limitations. This interaction is not significant *(p = 0.567)*, indicating that the relationship between *functional* limitations and life satisfaction does not vary significantly between urban and rural *hukou* holders. Model 5 tests a three-term interaction between *hukou* region, gender, and functional limitations. This interaction is also not significant *(p = 0.923)*, indicating that there are no significant differences between men and women in the moderating effects of *hukou* status.

## Discussion

This research used a nationally representative sample of Chinese older adults followed over four years to examine the association between functional limitations and psychological well-being. The analyses confirmed that functional limitations are associated with depression and life satisfaction when standard covariates were controlled, but these associations were substantially reduced when fixed-effects models were applied to eliminate all time-stable confounders. The reduction in the associations between functional limitations and psychological well-being in the fixed-effects models coheres with evidence of substantial confounding in the limitations-depression association in Western samples (Bierman, 2012). This pattern of evidence suggests that aging researchers should be aware that conventional multivariate modeling techniques that rely on observed controls are likely to fail to control for time-stable sources of spuriousness. Consequently, analyses are likely to overestimate the consequences of stress on psychological well-being, and this tendency appears to occur across geographic regions.

At the same time, the associations that remained significant even with these controls suggest that functional limitations, as chronic stressors, contribute to depression and a lower level of life satisfaction among older adults. The association with life satisfaction was relatively weak once time-varying and time-stable sources of spuriousness were taken into account, though, suggesting that the primary effects of functional limitations are on depression. The fact that we see these differences across outcomes also reinforces the importance of taking multiple aspects of psychological well-being into account when examining the effects of stress. In particular, it appears critical that future research on functional limitations takes both positive and negative indicators of psychological well-being into account as a means of presenting a full depiction of the extent of the psychological consequences of functional limitations.

We also hypothesized that rural *hukou* holders would experience more deleterious effects of functional limitations on psychological well-being. This hypothesis is partially confirmed. We found that the deleterious association between functional limitations and depression is stronger among older adults living in a rural *hukou* as compared to those living in an urban *hukou*, but *hukou* does not moderate the effects of functional limitations on life satisfaction among Chinese older adults. This finding supports the idea that the *hukou* system, as a determining social status in China, modifies the extent to which stressors influence psychological well-being, and therefore, plays an important part in fostering inequalities in psychological well-being among Chinese older adults. A stress process perspective suggests that the consequences of stressors for psychological well-being are differentiated by individual placement in structures of social stratification (Pearlin & Bierman, 2013). This perspective, therefore, sensitizes research to societal factors that stratify vulnerabilities to the effects of stress. Yet, research in a Western context mostly focuses on the roles that race, gender, and socioeconomic status play in stratifying the psychological effects of functional limitations (Lewis Brown & Turner, 2010; Margaretten et al., 2011; Noh et al., 2016; Parmelee et al., 2012; Schieman & Plickert, 2007). Longitudinal research on functional limitations and psychological well-being, for instance, suggests that women with functional restrictions are likely to have higher levels of depression than men (Noh et al., 2016). A higher level of socioeconomic status can mitigate the negative effects of functional limitations on psychological well-being (Schieman & Plickert, 2007). However, such appreciation of core stratifying agents may overlook the cross-cultural variation of alternative structuring agents that are unique to non-Western contexts. Our research, therefore, contributes to aging research by showing how research on the effects of stress in older adults must take into account how nation-specific stratifying factors may crucially differentiate the consequences of stress, especially when considering non-Western contexts that are structurally distinct from previous aging studies based in Western nations.

Yet, this research also does not find that *hukou* status modifies the effects of functional limitations on life satisfaction among Chinese older adults. Although this may suggest limitations in the degree to which *hukou* acts as a modifier for the effects of stress, it should also be kept in mind that the overall association between functional limitations and life satisfaction was relatively weak once multiple aspects of spuriousness are taken into account. There was, therefore, little effect of functional limitations on life satisfaction for *hukou* status to moderate. Thus, it appears that *hukou* status is an important modifier for the effects of stress, but only when there is a relatively distinct effect of stress to modify. Future research that examines the degree to which *hukou* status modifies the effects of additional stressors across multiple outcomes would be useful to further show whether a marked overall effect of stress is critical for the moderating effects of *hukou* to come into play.

Multiple factors may also help to explain why *hukou* status does not modify the effects of functional limitations on life satisfaction among Chinese older adults. Life satisfaction, by definition, is a cognitive evaluation of whether individuals’ social and relational needs are fulfilled (Headey et al., 1993; Lou, 2010). Rural lifestyle may benefit older adults’ life satisfaction by providing a strong social tie from kinship, and multigenerational residence in rural areas may also benefit life satisfaction for rural older adults (Gruijters, 2017; Liu et al., 2019; Shen & Yeatts, 2013). In addition, rural culture honors older people's wisdom and, therefore, may enhance life satisfaction for older adults (Tan & Barber, 2020; Zhang et al., 2016). The cultural factors in rural societies may contribute to a counterbalancing effect that masks the moderating effects of the *hukou* system on the psychological well-being of Chinese older adults. Even though rural people may have greater social ties to fulfill their social and relational needs, these resources do not result from the advantages engendered by higher social positions. Instead, the potential psychological benefits are provided by the cultural aspects that are rooted in rural China. More importantly, the limitations in the degree to which *hukou* acts as a modifier for the effects of stress do not mean that rural people are better off; it means that the inequality engendered by the *hukou* system is masked by the psychological “benefits” that seem to be provided to rural people. Future research that examines *hukou* status and psychological well-being should consider the cultural differences between urban and rural societies and unmask the urban–rural inequalities that underlie individuals’ psychological well-being.

We also do not observe significant differences between men and women in the moderating effects of *hukou* status on either outcome. This lack of differences by gender may be due in part to an age-related decline in gender differences as people age (Ferraro & Wilkinson, 2013), as well as increases in “socioemotional selectivity” (Blazer & Hybels, 2005, p. 1249), which further narrow the gender differences in reactions to stressors. More importantly, people with disadvantaged social statuses can develop strategies to protect themselves from the deleterious consequences of being disadvantaged (Louie & Wheaton, 2019). Women in China have experienced severe gender inequality as they are marginalized in the labor market and denied rights such as land possession and household ownership (Hershatter, 2007; Ji et al., 2017; Li & Wu, 2011; Li et al., 2008). With all the life vicissitudes and scarce resources experienced over the life course, Chinese women may use available resources more wisely or purposefully to seek more help, thereby challenging our expectations of gender differences in the moderating effects of *hukou*.

The current study also has political implications. Although the well-being of rural people is recognized by recent policymaking, these policies tend to focus on somatic illness (Xu et al., 2016). Psychological well-being among rural older adults is neglected in political intervention. This study can draw attention to the psychological well-being of the marginalized groups in China and help the government reduce the health inequalities engendered by the dualistic rural–urban structure. To address inequalities in psychological well-being among Chinese older adults, policymakers should pay more attention to older adults with rural *hukou*, and interventions should be more targeted and specific. For example, facilities and services should be delivered toward rural areas and vulnerable populations. Deeper and more systematic reform is needed to reduce the health inequities caused by the *hukou* system. The current social welfare schemes should be further improved to reduce urban–rural disparities and therefore make the benefits more comprehensive.

Although this research expands the study of stress and aging, weaknesses also should be noted. First, the measure of *hukou* did not take locality into account. In addition to the rural–urban division of *hukou*, Chinese citizens are also distinguished by the possession of a local *hukou* (Chan, 2019). Social welfare and services differ by administrative units in China; the locality of one's *hukou*, therefore, may further determine her eligibility for social welfare and services (Chan & Wei, 2019). However, given that the major difference in rights and eligibility is between rural and urban *hukou* (Kuang & Liu, 2012), and only a small number of people have changed their locality of *hukou* (Chan, 2012), the rural–urban division of *hukou* status plays a more crucial role in moderating the effects of stressors, compared to the locality of *hukou*. Second, this research only examined two indicators of psychological well-being, overlooking the impact of functional limitations on other psychological well-being outcomes, such as anxiety or alcohol abuse. Given the importance of considering the extent of the psychological consequences of stress, future studies of the psychological effects of functional limitations should take additional outcomes into account. Furthermore, life satisfaction was an ordinal measure. Although we use standard errors that have been shown to be robust to deviations from normality in ordinal measures when there are at least five response levels (Rhemtulla et al., 2012), additional research that uses a measure with more opportunity for variance, such as the ten-category measure used in the World Values Survey, may detect more subtle changes in life satisfaction over time.

## Conclusion

This research suggests that functional limitations are associated with deteriorated psychological well-being among older adults in China. However, this association is likely to be overestimated in analyses that do not consider the unobserved time-stable sources of spuriousness. Further, this research suggests that even though functional limitations have substantial consequences for psychological well-being, *hukou* status, as a core stratifying agent in China, can still moderate these effects. This research, therefore, calls attention to the specific stratification factors that may crucially foster inequalities in psychological well-being among older adults, particularly the way that a non-Western context may contain additional stratifying factors not observed in Western studies of aging.

## Notes

1. The poverty lines for 2011, 2013, and 2015 are 2300, 2736, and 2855 Chinese Yuan, respectively. This infomation is adapted from China, D. o. H. S. N. B. o. S. o. (2016). *Poverty Monitoring Report of Rural China*. C. S. Press.
